# Conservative and surgical treatment of the chronic Charcot foot and ankle

**DOI:** 10.3402/dfa.v4i0.21177

**Published:** 2013-08-02

**Authors:** Mehmet Fatih Güven, Atakan Karabiber, Gökhan Kaynak, Tahir Öğüt

**Affiliations:** Cerrahpasa Faculty of Medicine, Department of Orthopaedics and Traumatology, Istanbul University, Istanbul, Turkey

**Keywords:** Charcot foot, diabetes mellitus, total contact cast, arthrodesis, diabetic neuropathy

## Abstract

Charcot neuroarthropathy (CN) is a severe joint disease in the foot and ankle that can result in fracture, permanent deformity, and limb loss. It is a serious and potentially limb-threatening lower-extremity late complication of diabetes mellitus. The aim of this manuscript was to evaluate modern concepts of chronic CN through a review of the available literature and to integrate a perspective of management from the authors’ extensive experience.

Charcot neuroarthropathy (CN) was first described by neurologist Jean-Martin Charcot in 1868 in a group of patients with syphilis, but the mystery of pathophysiology remains (1–3). CN is considered as a destruction of bones and joints secondary to underlying neuropathy, trauma, and perturbations of bone metabolism. Although CN is a clinical diagnosis, recent advances in diagnostic imaging have eased the clinical challenge of deciphering infection from Charcot changes (1–3). Limb salvage is a new option for advanced medical and surgical treatment. Pharmacologic therapies have also shown promise for treatment (1, 2).

CN is a progressive, non-infectious neuro-osteoarthropathy of the bones and joints in patients with sensorial neuropathy leading to destruction of the foot architecture (1, 2). CN is an inflammatory condition that leads to osteolysis and is indirectly responsible for the progressive fractures and multiple joint dislocations that characterizes its presentation. It commonly presents in the midfoot but also occurs in the forefoot and hindfoot. CN also has been associated with autonomic neuroarthropathy, infection (leprosy, HIV), toxic exposure (ethanol, drug related), rheumatoid arthritis, multiple sclerosis, congenital neuropathy, tabes dorsalis, traumatic injury, metabolic abnormalities, and syringomyelia (3, 4). However, diabetes mellitus has become the most common etiology in recent years. The precise incidence of CN in people with diabetes mellitus had been previously estimated to be between 0.1 and 0.4%, but recently the incidence was estimated to be 0.08–0.13% (1, 5, 6). In CN with foot and ankle deformity involvement in diabetes and neuropathy, the incidence is up to 7.5%, and more than 9% of those have bilateral involvement (5, 6). In addition, its prevalence is less than the actual numbers because of misdiagnosis or delay in diagnosis (1).

The diagnosis of CN has been reported to be delayed because of misdiagnosis such as gout, deep vein thrombosis, soft tissue injury, rheumatoid arthritis, or infection (7–10). The consequences of the delay in diagnosis are severe and debilitating such as structural deformity of the foot (9, 10). This subsequent deformity in the presence of peripheral neuropathy greatly increases the risk of skin ulceration and lower limb amputation (10–12). CN is a medical emergency, because if it is diagnosed earlier, the treatment can prevent the destructive process (9, 10, 13).

The acknowledgement of the pathogenesis of CN is important for deciding the treatment strategy. The pathogenesis of the disorder is not well understood, and there is no consensus on the pathologic process that causes CN (1). Therefore, it is likely that pathogenesis of this disease is multifactorial (1, 10). The pathogenesis of CN has been explained by two major theories. Neurovascular theory implies that joint destruction is secondary to an autonomic stimulated vascular reflex causing hyperemia and periarticular osteopenia with contributory trauma. On the other hand, the neurotraumatic theory suggests that CN is an overuse injury in which insensate joints are subjected to either repetitive microtrauma or single traumatic event that leads to typical Charcot changes (1, 2, 10, 12). Lack of protective sensation delays the recognition of bone injuries that may overload the insensate limb and leads to an active Charcot process (13–15). Sensation loss prevents the affected individual from adopting normal protective mechanisms, specifically offloading and activity modification, and from seeking medical attention (1, 13–15). Diagnostic clinical findings include autonomic dysfunction, components of neurological, vascular, musculoskeletal, and radiographic abnormalities.

In chronic CN, the patients’ symptoms of warmth and swelling are decreased, and inflammation is usually not present. On radiographic evaluation, osteophytes, joint consolidation, and arthrosis are the findings of chronic CN. Dislocation of the tarsometatarsal joint with break in the talo-first metatarsal line and reduced calcaneal inclination angle can be seen by the lateral radiograph in a late stage of chronic CN deformity. Deformity begins with the medial column and proceeds to the lateral column in the late stages of CN. Abduction of the foot arch and development of bony prominences lead to deformity and ulcerations of the foot. The rocker-bottom foot deformity, with or without plantar ulceration, indicates a severe chronic deformity that is typical for the chronic CN (7, 8, 15) (Fig. 1). The rocker-bottom deformity begins with the collapse of naviculocuneiform joint which is involved in the naviculocuneiform pattern. The perinavicular pattern begins with the osteonecrosis or fracture of the navicular. In the late stages, the talus is completely dislocated from the navicular and ulceration of the calcaneocuboid interval begins.

**Fig. 1:**
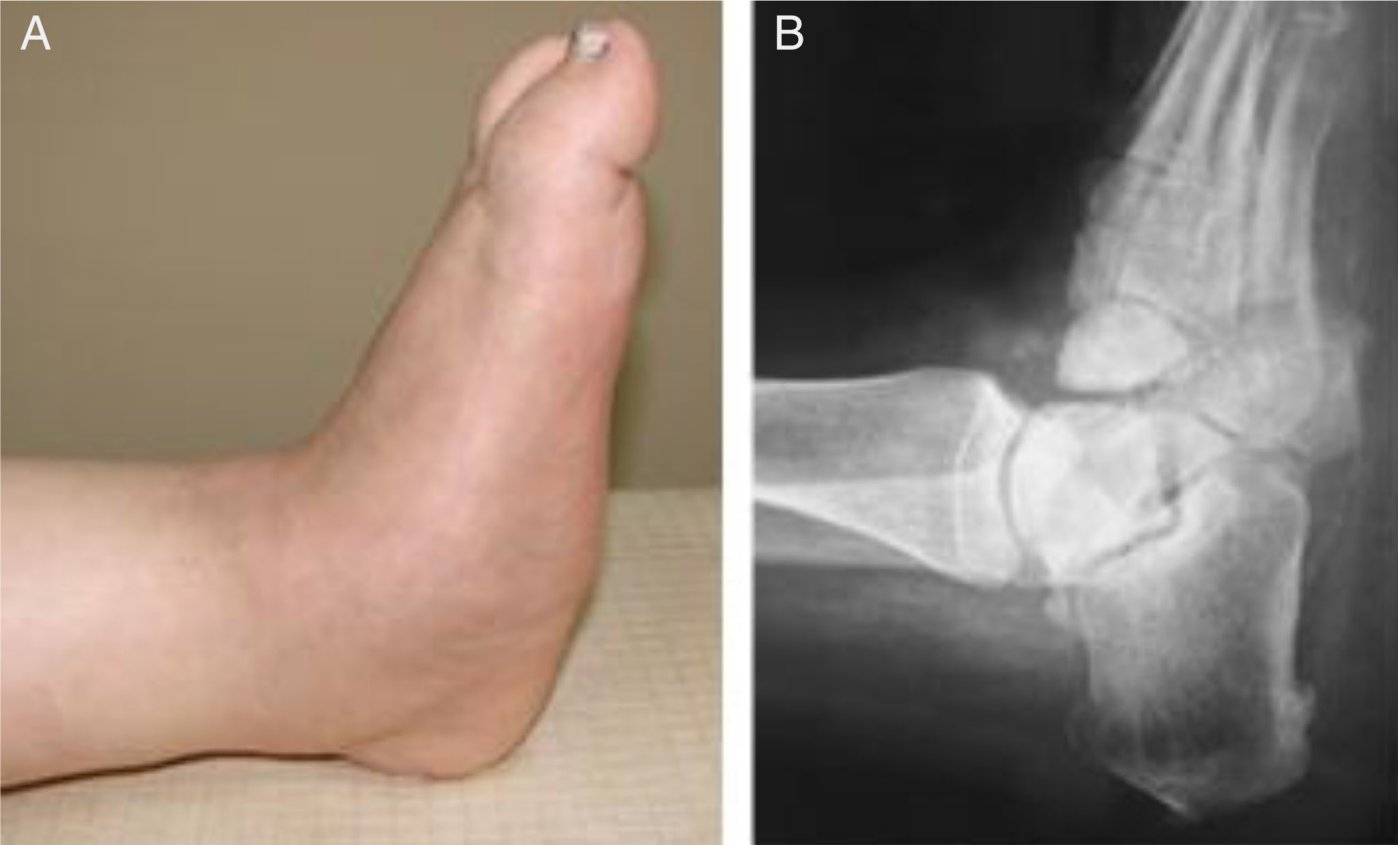
Rocker-bottom Charcot foot (a) Clinical appearance (b) Lateral radiograph.

## Stages of Charcot foot

Numerous systems have been proposed to classify the Charcot foot. Eichenholtz proposed a systematic classification based on radiological findings in 1966 (16). His grading was mostly a radiological evolution of the condition through time and did not include clinical manifestations (1, 16). Although he described 3 stages, a ‘Stage 0’ is defined by a hyperthermic, usually painful and swollen foot where no radiographic changes are identified, but magnetic resonance imaging show bone marrow edema (17–19). The clinician may often suspect either deep infection or cellulitis (20–22).

The classical staging system as proposed by Eichenholtz is improved in time and supported with clinical manifestations as follows: Stage I is the fragmentation phase (acute stage) where plain radiographs demonstrate osteopenia, periarticular fragmentation, and subluxation or frank dislocation of joints. Clinically, the foot continues to be warm and edematous and may demonstrate increased ligamentous laxity (1, 16); Stage II, coalescence stage, subacute Charcot (reparative stage) represents the early healing phase. Edema and warmth decreases and absorption of debris, fusion of bony fragments, new bone formation, and/or sclerosis of bone ends are other typical changes (1, 16); and Stage III, consolidation stage, chronic Charcot (reconstructive or bone healing stage) is characterized by an absence of inflammation and progression to a more stable, although often deformed foot or ankle (1, 16).

Brodsky described an anatomical classification based on the four areas most commonly affected by CN (23, 24). Brodsky Type I (midfoot) includes the naviculocuneiform and metatarsocuneiform joints, which is the most common (60%) (23, 24). The second most common type, type II (hindfoot) includes the subtalar, talonavicular, or calcaneocuboid joints (23, 24). Type II approximately accounts for 30–35% of anatomical incidence. Type III is divided into ‘A’ and ‘B’. Type IIIA involves the tibiotalar joint and associated bones, and type IIIB involves a pathologic fracture of the tuberosity of the calcaneus.

Existing classification systems for Charcot foot are predominantly based on radiographic findings and anatomical locations. Thus, these systems are insufficient in identifying the disease in early stages, providing prognostic data or direct the clinician to specific treatment options. New staging systems based on clinical findings that also qualify a Charcot patient as active or inactive, according to the status of the inflammation, should be highly considered.

## Management of the Charcot foot

The goal in the treatment of CN is to achieve a plantigrade, stable foot that is able to fit into a shoe and to also prevent a recurrent ulceration. Treatment depends on many factors including the location, phase of the disease process, deformity, presence or absence of infection, and the other comorbidities (1). The severity of the disease decides the goals which need to be specific and realistic to achieve (1, 25) while the treatment plan can vary from basic shoe modification to limb amputation.

Recommended treatment for Eichenholtz stage 0 is frequent follow-up with serial radiographs to monitor the development of Stage I CN and patient education on diabetic foot care (1, 2, 16, 26). Eichenholtz stage I CN is successfully treated with immobilization and non-weightbearing in a total contact cast (1, 2, 16, 27, 28). The duration of the treatment is usually determined according to the practitioner's opinion that involved joints will be able to sustain physiologic stresses. In this phase, frequent follow-up and radiographic evaluation with serial casting is very important until erythema, color, and inflammation has resolved (1, 2, 16, 22). Stage II (coalescence-subacute phase) is typically treated with protected weight bearing with a total contact cast or a molded total-contact polypropylene ankle-foot orthosis (7, 22). In Stage III (reconstruction-chronic), if the foot is plantigrade, the patient can use custom inlay shoes (21, 22, 25). If the patient has a non-plantigrade foot or recurrent history of ulcerations, debridement, exostectomy, correction, or fusion with internal fixation may be an option. Also, in Stage III with the presence of osteomyelitis, recommended treatment is surgical debridement with or without staged reconstruction with internal or external fixation, or amputation (1, 2, 10, 17, 21, 22, 29).

## Non-surgical treatment for the Charcot foot

The non-surgical treatment of CN is usually considered for the acute stages and includes offloading of the involved foot, treating the bone disease, and preventing further fractures and/or dislocations (2, 10, 22, 25, 30, 31). In addition, this treatment can also be used in certain chronic CN patients and foot ulcerations (31). Offloading is the most important step in the management strategy of acute CN for consolidating the progression of deformity (1, 2, 16, 27, 28). Total contact casting (TCC) has been considered as the gold standard in the treatment of neuropathic diabetic plantar foot ulcers (1, 2, 17). The utilization of a TCC reduces the mechanical forces, inflammation, and edema; redistributes the plantar pressure; limits bone and joint destruction; and can consolidate the progression of deformity (1, 2, 7, 20, 23, 25). Its overall aim is to maintain a plantigrade foot which can then allow weightbearing in a shoe or brace (30).

**Fig. 2:**
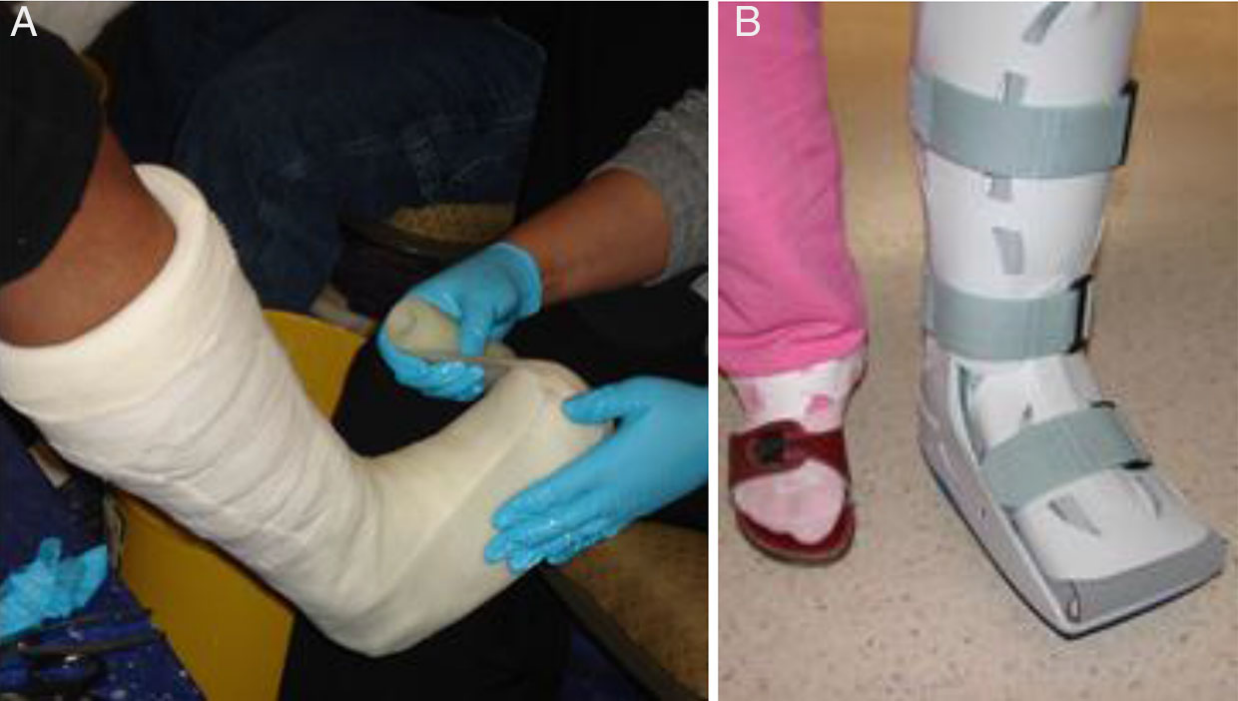
(A) Total contact cast application and removable walker brace usage (B).

TCC should encase the entire foot and ankle, with all major bony prominences well-padded with cotton-based bandages (Fig. 2a). Frequent cast changes are critical in reducing complications because setting can lead to instability and ulceration within the cast (1, 2, 10, 22), and patients should be closely monitored on a weekly basis. At each visit, the cast or device should be inspected and removed. Wounds should be inspected, if necessary sharply debrided, measured, and photographed (30, 32–35). When the active phase has ended, the patient can be fitted with Charcot restraint orthotic walker and later, with a custom shoe or orthoses (33–35). The average cast duration in chronic CN with ulceration is 5 weeks, with progression to therapatic footwear at 12 weeks. Some patients may need a cast for over a year and complications may include simple skin macerations (34, 35).

Consequently, patients with chronic Charcot foot ulcers can be treated with TCC or removable walker braces (23, 25). In some studies, removable walker braces have been used successfully to treat acute or chronic ulcerated CN (33–35) (Fig. 2b). Removable walker braces have also been effective in reducing plantar foot pressures and treating diabetic foot ulcers (33–35). Low cost of the removable walker braces compared with the multiple TCC changes makes them an acceptable treatment option (33, 34). Disadvantages of removable walker braces include the inability to fit severe deformities and potentially limited compliance (33–35).

## Pharmacologic therapy for chronic CN

In CN, available treatment options are based on the balance between bone resorption and formation (36). There is firm evidence that CN is associated with increased osteoclastic activity, and antiresorptive therapies have been used with some success. Bisphosphonates and calcitonin have been used in the treatment of CN (1, 2, 36). Bisphosphonates can inhibit osteoclastic bone resorption, so they are usually used in treatment of conditions characterized by abnormal turnover, especially acute active phase of CN and sometimes in chronic phases (36–39). Some of the patients with CN may not tolerate oral bisphosphonates but may benefit from intravenous therapy of pamidronate or zolendronic acid (36). Pamidronate is most commonly used and it acts on hydroxyapatite crystals in newly synthesized bone matrix, blocking access of osteoclast precursors to the matrix (39, 40). Pitocco et al. showed significant reduction in bone resorption markers with the use of another bisphosphonate alendronate and clinical improvements in CN foot at 6 months (38, 39). Intranasal calcitonin is another antiresorptive agent that has been studied in the treatment of CN (40). This therapy has shown fewer complications compared to bisphosphonate use (36–40). However, there is little evidence to guide the use of available pharmacological therapies to promote the healing of CN. Most pharmacologic agents still remain theoretic, with most studies evaluating only secondary clinical markers (20, 36, 37, 40–42).

## Surgical treatment for chronic CN

Surgical treatment of CN of the foot and ankle is first and foremost dependant on the physician's opinion. Patient's medical comorbidities, compliance, location and severity of deformity, presence or absence of infection, pain or instability are the factors considered in the decision of surgical treatment. In chronic CN, among the surgical techniques of realigning and stabilizing the deformed diabetic Charcot foot, well-known are Achilles tendon lengthening, plantar osteotomy, osseous debridement, realignment osteotomy, selective or extended arthrodesis, and open reduction with various forms of internal fixation with or without external fixation (43). There are no comparative studies that have been made between the surgical choices of CN.

In recent studies, the advantages of earlier surgical correction of deformity and arthrodesis have been proposed, which are based on the assumption that surgical stabilization would increase a patient's quality of life (43). Recurring ulcers, joint instability, pain, associated malalignment, prominent exostosis, and potential skin complications from inability to brace or from a non-plantigrade foot are the most important surgical indications in chronic CN. Due to the increased risk of wound infection and mechanical failure of fixation, surgery should be avoided during the active inflammatory stage. Series by Mittlmeier et al. showed the advantages of early correction of deformity combined with arthrodesis (44). Early surgical series showed improvement in restoring a plantigrade foot and preventing recurrence of ulceration, although non-union, failure, and loss of initial correction were common. CN patients who undergo minor or major surgical procedures have ranged from 14 (45) to 51% (46, 47). According to a study by Saltzman et al. (48), amputation rates of the lower extremity have ranged from 3 to 9% depending primarily on avoidance of ulceration.

## Exostectomy and Achilles tendon lengthening

**Fig. 3:**
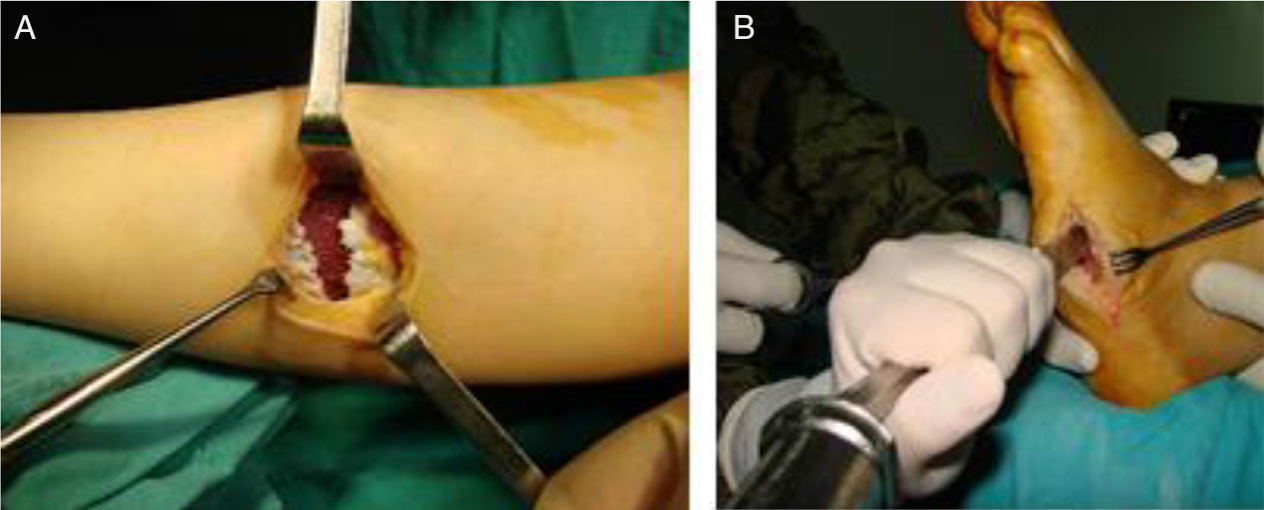
A 58-year-old female with a Stage III diabetic chronic Charcot neuroarthropahty treated with a gastrocnemius lengthening (a) and exostectomy (b).

If ulceration occurs after failed conservative treatment, exostectomy of an ulcer-inciting bony prominence can be consideredBrodsky and Rouse's studies indicated that limb-salvage rates reached 90% with exostectomy (24). Exostectomy techniques have been performed successfully in many studies on surgical treatment in Charcot midfoot deformities and ulcers (49, 50). In Laurinavicience's study which consisted of 20 patients, it has been reported that exostectomy procedures were effective and safe for treating Charcot midfoot deformities and ulcerations (50). Exostectomy has been used successfully in relieving bony pressure that cannot be accommodated with orthotic and prosthetic measures. Generally, these prominences are wrongly considered as a new bone formation, but actually those are tarsal bones that have shifted into a non-anatomic position, leading to chronic ulcerations. After exostectomy and protective bracing, if it is necessary, antibiotic therapy could be useful for a successful result. Even when exostectomy is performed for a tarsometatarsal deformity (Brodsky type 1), Achilles tendon lengthening should also be considered for the patient with concomitant recurrent plantar ulceration and severe equinus contracture. Lengthening of the Achilles tendon or gastrocnemius tendon recession can decrease the forefoot pressures and improve the alignment of the ankle and hindfoot to the midfoot and forefoot (Fig. 3). Catanzariti et al. reported that Achilles tendon lengthening, in addition to osteotomy in CN patients with lateral midfoot ulcers, healed primarily with a rate of 38% (49). However; in the same study, the rate of primary healing in medial midfoot ulcers was 92% (49). A randomized study by Mueller et al. showed that in patients with CN and midfoot deformity, TCC with Achilles tendon lengthening were 75% less likely to have ulcer recurrence at 7 months (51, 52). Their recommendation was to consider an Achilles tendon lengthening in patients for equinus contracture and concomitant recurrent plantar ulceration (51, 52).

## Arthrodesis

**Fig. 4:**
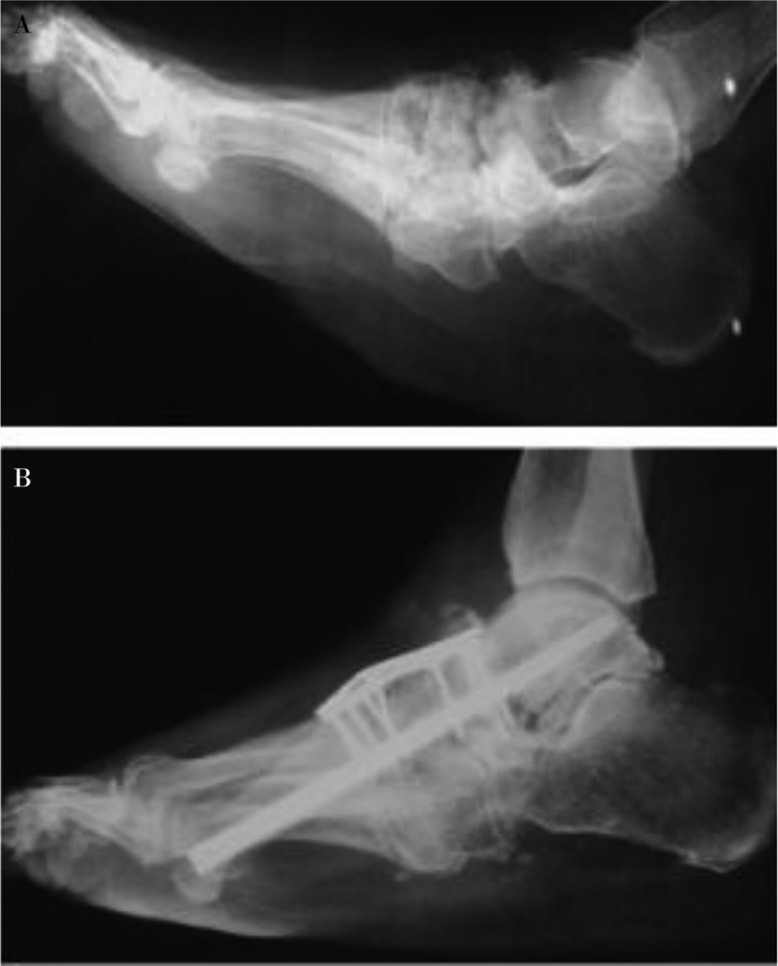
A 62-year-old female with a 10-year history of diabetes mellitus and Stage III chronic Charcot neuroarthropathy. Preoperative lateral radiograph (a) and postoperative lateral radiograph (b) showing the arthrodesis with internal fixation after osteotomy and deformity correction.

Arthrodesis can be a useful treatment option in patients with instability, pain, or recurrent ulcerations that have failed conservative therapy, despite a high rate of incomplete bony union (53–58). In chronic CN with more severe deformities, fusion may be the only alternative treatment option instead of a limb amputation (58–60). Internal or external fixation techniques can be used for arthrodesis procedures (Fig. 4). CN of the hindfoot and ankle results in higher complications than midfoot CN because of their high instability rates (55).

### Arthrodesis with internal fixation

Effective internal fixation techniques consist of pin, screw, nail, and plate fixation. Correction of deformity by midfoot osteotomy-arthrodesis, triple arthrodesis or tibio-talo-calcaneal arthrodesis, depending on the level of deformity has been described by Shibata et al., Pinzur et al., Saltzman, and Stone (17, 46–48, 58, 59). These can be single or staged procedures, based on the presence of infection and may require osteotomy with autografting. If there is a suspicion of infection, staged procedures may be required than a single stage surgery (61–63). Therefore, the type of the surgical approach depends on many factors, including the severity and location of deformity, stability, and presence or absence of infection. Intramedullary nails can be used successfully for internal fixation of chronic CN treatment. In the series by Caravaggi et al., tibiocalcaneal intramedullary devices offer an alternative for Brodsky type 2 (involves the subtalar and/or the Chopart joints) and type 3 (involves the ankle joint) deformities (61). Most fusion techniques usually require a brace or cast with or without partial weightbearing for 3 months, which is mostly followed by prolonged or permanent protective bracing (58, 59, 62). There are some case series involving arthrodesis for limb salvage that have resulted in relatively successful results. The study by Caravaggi et al. included 45 diabetic patients who underwent unilateral ankle arthrodesis for CN before the development of ulceration and bone infection, and after a 5-year follow-up, 39 patients (86.67%) returned to independent ambulation wearing (61).

Simon et al. reported successful results of 14 patients with midfoot CN, who were treated with anatomic reduction and primary arthrodesis (57). All 14 patients were treated with adequate anatomical reduction and primary arthrodesis, and all achieved a successful clinical outcome (57). Lowery et al. (64) reported a systematic review of surgery for Charcot. Among the 246 reported patients, 76% achieved bony fusion, 22% developed fibrosis or non-union, and 1.2% underwent amputation (64). Most non-unions are sufficiently stable to allow mobilization with bracing. However, most of these studies had a bias including inadequate number of patients and involving different anatomical sites for foot and ankle arthrodesis (47, 53, 55, 56, 65).

Timing of surgery has traditionally been reserved for Eichenholtz coalescence or reconstruction phases. Nonetheless, the timing of surgery remains controversial due to lack of high-level evidence-based literature on reconstructive surgery in CN (64). In the literature, most series are small, with short to intermediate follow-ups (34, 37, 48–50), and in most of the series, complication rates are high (47, 53, 55, 65). Infection, hardware malposition requiring removal, recurrent ulcerations, and fractures are shown to be common. Although, in recent studies, 36–100% patients had achieved bony union, a stable fibrous union can be acceptable with adequate bracing (47, 53, 55, 56, 65). If an unstable fibrous union or uncontrolled infection occurs, amputation may be the only treatment option.

### Arthrodesis with external fixation

In recent years, external fixation has become more popular, because of being a less invasive treatment option for chronic Charcot foot deformities. This technique has some advantages such as being a single-stage treatment in the presence of osteomyelitis or ulcerations, ease of monitoring of soft tissue healing, decreased amount of soft tissue dissection and decreased operative time (54, 56). Also, open wounds may be monitored and treated easily by this technique. Ulcers with underlying osteomyelitis, poor soft tissue envelope, poor bone quality, and morbid obesity are some of the indications for external fixation. The surgeon can avoid placement of hardware in infected areas or open ulcerations. Benefits of external fixation include the minimal invasive technique, allowing gradual and accurate anatomic realignment of dislocated or subluxated joints, and the limited neurovascular compromise because of the gradual and slow correction. Good results have been reported with external fixation techniques in patients who were not suited for internal fixation and otherwise may have required amputation (45, 46, 54, 56). In some studies, external fixator treatment limb salvage rates are over 90% and recurrent ulceration was rare (45, 46, 54, 56) with most common complication being a pin-tract infection (45, 46, 54, 56). Pin-tract infections can be avoided by patient education and compressive dressings. If pin or wire insertions show signs of irritation, the surgeon can adjust the location of the pins and wires or may also remove them, if necessary. Another disadvantage of external fixation is the long learning curve. Using extensive hardware as an external fixator must be reconsidered, as postoperative evaluation of the bone and joints will be difficult to examine on plain radiographs, and it also must be kept in mind that hardware may not be tolerated by the patient secondary to cosmetic problems and damage caused to the contralateral limb by the hardware.

**Fig. 5:**
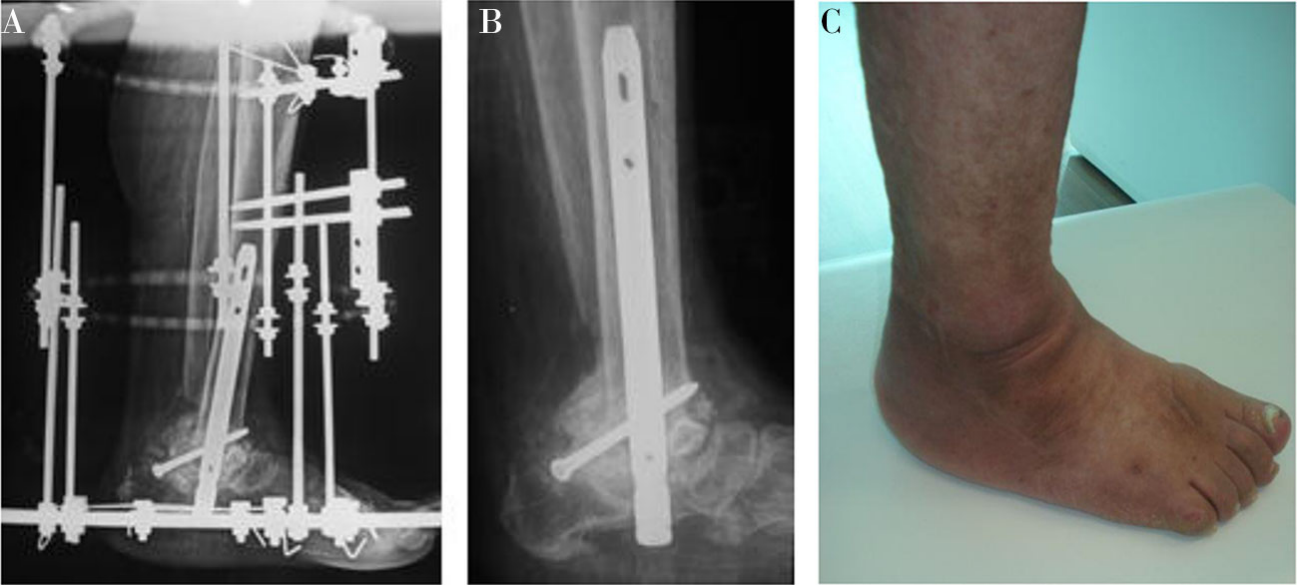
Postoperative lateral radiograph (a) showing the combined utilization of internal and external fixation, followed by external fixation removal (b) and clinical outcome 4 months after the surgery (c).

Usage of multiplane circular external fixation decreases the rates of the need for extensive surgical exposure in Charcot foot and ankle reconstruction, and it is also beneficial for reducing deformities while maintaining the reduction during consolidation. It can also be used for improving fixation with open reduction and primary arthrodesis techniques (Fig. 5). Arthrodesis with external fixation has been described by Cooper et al., Zarutsky et al., Zgonis, Farber et al., Pinzur et al., and the many other experts with successful results (45, 46, 54, 56, 66–70). Experts usually recommend deformity correction via single or multiple wedge osteotomies and primary arthrodesis with the application of an external circular multiplane fixator in severe deformities for long-term successful results (56, 66–70). The study by Pinzur (46) introduced a treatment option for non-plantigrade midfoot chronic Charcot deformity using a neutral positioned ring external fixator. This simple neutralization frame was used on 26 Charcot feet in 26 patients after deformity correction through limited incisions and other adjunctive procedures, such as tendon lengthening (46). He reported a 92% favorable outcome in 26 patients who underwent reconstruction for a high- risk, non-plantigrade Charcot midfoot deformity with a ring fixator.

In a study by Cooper et al., limb salvage rate was 96% in 83 patients with CN treated with static and dynamic ring external fixation (53). The average follow-up was 22 months. Three of the patients required amputation because of uncontrolled infection or unstable pseudoarthrosis. Also, there were only two recurrent ulcers. Numerous studies have reported success with arthrodesis of the Charcot midfoot deformity with fusion rates ranging from 78 to 100% (45, 46, 54, 56, 66–70).

In addition to external circular multiplanar fixator treatment, procedures like ulcer resection, biopsy, wedge osteotomy, soft tissue coverage via local muscle, or distant pedicle flaps can be used in the presence of an ulcer or osteomyelitis (65–69). The goal of these procedures is to provide better changes for a shoe or brace fitting with a decreased possibility of ulcer recurrence and instability.

### Arthrodesis of advanced Charcot foot deformity with combined technique

In severe Charcot foot deformity, reduction should be done gradually with external fixation (70). A stable Charcot foot deformity usually requires osteotomy for correction whereas in an unstable or an incompletely coalesced Charcot foot, correction can be obtained with gradual distraction without osteotomy (70). Recently, even if the radiographic appearances show coalescence, most Charcot deformities can be corrected with distraction, without osteotomy (70). In this technique, the first step is osseous realignment by external circular multiplane fixator using the principles of ligamentotaxis. After external fixation removal, the second step is arthrodesis of the affected joints by percutaneously inserted intramedullary metatarsal screws. With this technique, the main goal is to achieve gradual relocation of the forefoot on the hindfoot. Correction with external fixation is minimally invasive and allows gradual accurate anatomic realignment of the dislocated/subluxed Charcot joints without loss of foot length or bone mass and provides partial weightbearing. Weightbearing starts gradually, and this treatment is usually completed in 4 to 5 months (66–70).

In the midfoot CN, tarsometatarsal instability is treated by primary arthrodesis with additional allogenic bone graft material. Also, autogenous platelet-rich plasma and bone growth electrical stimulation can be used to increase the success rate of joint fusion. In the rearfoot/ankle CN, the most common primary arthrodesis techniques are multiplane circular fixators, which include triple, pantalar, and talectomy with tibiocalcaneal or concomitant usage of tibionavicular and calcaneocuboid arthrodesis.

## Amputation

**Fig. 6:**
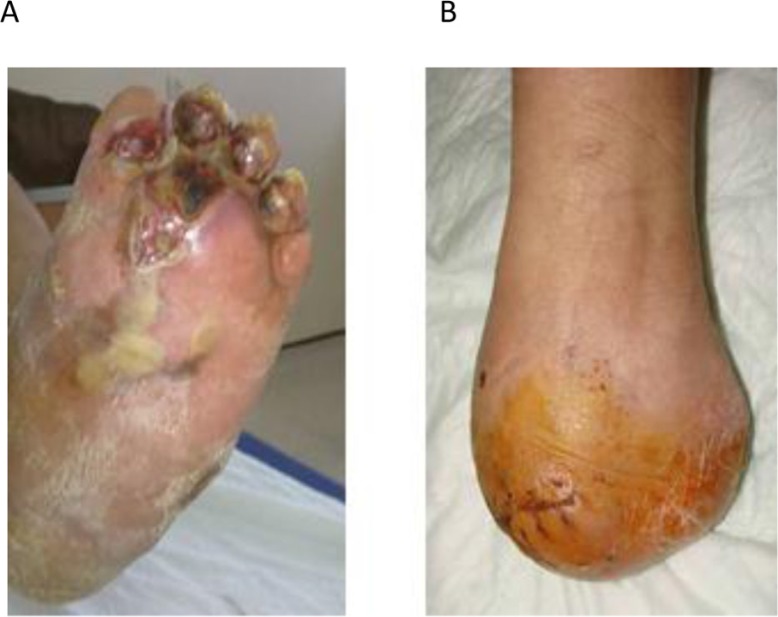
A 64-year-old male with a 20-year history of diabetes mellitus and bilateral chronic Charcot neuroarthropathies with a recurrent plantar ulceration and soft tissue necrosis (a) and subsequent Boyd's amputation (b).

Limb amputation is done only on the failure of previous surgery, and it is performed because of unstable arthrodesis or recurrent ulceration or infection (55, 58). Despite the advancement in wound treatment and antibiotic therapy, sometimes the ulcers and infections are very difficult to treat when they have spread to deep structures. At this stage, all treatment efforts may possibly be ineffective and major amputations may be inevitable (71). For many years, many surgeons believed that major amputation was the appropriate treatment option for complicated late stage Charcot foot deformities with concomitant infection. In the past 10 years, this opinion changed significantly. With advancement in surgical techniques and wound treatment strategies, amputation rates significantly decreased. In a retrospective review of CN deformity patients, Saltzman et al. reported that amputation rate was 2.7% (48). The surgeon should consider many factors to determine whether to perform primary amputation or limb salvage techniques. Limb amputation is especially reserved for those patients who are incapable of tolerating a complex surgery or extended periods of non-weightbearing and may be unlikely to ambulate even with other surgical procedures (1). Amputation could be performed from various levels of the affected limb. Trans-tibial amputation is usually preferred, as prosthetic fitting is easier. However, hindfoot amputations such as the Syme procedure allow weightbearing mobilization on the stump, which can be useful for patients with generally poor mobility and often poor eyesight. Also, in recent years, Boyd's operation can be performed successfully in the late stage Charcot foot deformities which are complicated with infection and severe wound problems (71) (Fig. 6). Talectomy and calcaneotibial arthrodesis procedure was first described by Boyd (72). In this technique, the surgeon should first prepare the dorsal and plantar flaps before the ligamentous resection and talus excision (71). After removal of the talus, all joint surfaces are prepared before the calcaneus is fixed to the tibia. Finally, the arthrodesis site is covered by the musculocutanous flaps (71, 72) with the aim of providing a functional amputation stump. Boyd's operation results in about 5 cm shortness of the extremity and fusion between the tibia and calcaneus (71). After surgery, when the healing process is completed, patients are allowed to walk for short distances with accommodative custom made shoes (71).

## Conclusion

In the era of evidence-based medicine, CN of the foot and ankle remains a poorly understood disease, although recent clinical and basic science researches have improved our level of knowledge regarding its etiology and treatment. CN is a major complication of diabetes mellitus. It often presents without warning and can rapidly deteriorate into severe and irreversible foot deformity leading to an ulceration and amputation. The uncontrolled cycle of inflammation leads to foot and ankle destruction and severe deformities. Offloading is the most important initial recommendation for the treatment. Most patients with CN can be treated with immobilization and protected weightbearing. Utilization of a TCC is the preferred method of a non-surgical management. The overall benefit of the antiresorptive therapies on healing remains unclear, and the benefit of anabolic therapy with parathyroid hormone is yet to be established in chronic Charcot foot treatment. Although non-operative treatment with use of a TCC followed by an appropriate bracing and footwear is considered to be the gold standard treatment for CN, surgical treatment is essential when conservative treatment fails. Surgical treatment is reserved for chronic recurrent ulcerations, unbraceable deformity, acute fracture, dislocation or infection. Surgical intervention includes exostectomy, Achilles tendon lengthening, and arthrodesis. Arthrodesis can be performed with internal fixation and can be combined with external fixation simultaneously or just with only external fixation. Also, additional osteotomies may be used. Regardless of the chosen treatment pathway, all treatment protocols should be adjusted according to the patient based on their lower limb pathology, overall medical status, and ability to comply with treatment.

## References

[1] van der Ven A, Chapman CB, Bowker JH (2009). Charcot neuroarthropathy of the foot and ankle. J Am Acad Orthop Surg.

[2] Armstrong DG, Peters EJ (2002). Charcot's arthropathy of the foot. J Am Podiatr Med Assoc.

[3] Sanders LJ, Fryberg RG, Bowker JH, Pfeifer MA (2007). The Charcot foot. Levin and O'Neal's the diabetic foot.

[4] Miller DS, Lichtman WF (1955). Diabetic neuropathic arthropathy of feet; summary report of seventeen cases. AMA Arch Surg.

[5] Sinha S, Munichoodappa CS, Kozak GP (1972). Neuro-arthropathy (Charcot joints) in diabetes mellitus (clinical study of 101cases). Medicine.

[6] Klenerman L (1996). The Charcot joint in diabetes. Diabet Med.

[7] Nielson DL, Armstrong DG (2008). The natural history of Charcot's neuroarthropathy. Clin Podiatr Med Surg.

[8] Pakariennen TK, Laine HJ, Honkonen SE, Peltonen J, Oksala H, Lahtela J (2002). Charcot arthropathy of the diabetic foot. Current concepts and review of 36 cases. Scand J Surg.

[9] Chantelau E (2005). The peris of procalcitonin: effects of early vs. delayed detection and treatment of incipient Charcot fracture. Diabet Med.

[10] Perrin BM, Gardner MJ, Suhaimi A, Murphy D (2010). Charcot osteoarthropathy of the foot. Aust Fam Physician.

[11] Pecoraro RE, Reiber GE, Burgess EM (1990). Pathways to diabetic limb amputation. Basis for preventation. Diabetes Care.

[12] Reiber GE, Vileikyte L, Boyko EJ (1999). Causal pathways for incident lower extremity ulcers in diabetes from the two settings. Diabetes Care.

[13] Tan AL, Greenstein A, Jarrett SR, McGonagle D (2005). Acute neuropathic joint disease: medical emergancy?. Diabetes Care.

[14] Eloesser L (1917). On the nature of neuropathic affections of the joints. Ann Surg.

[15] Salo PT, Theriault E, Wiley RG (1997). Selective ablation of rat knee joint innervation with injected immunotoxin: a potential new model for the study of neuropathic arthritis. J Orthop Res.

[16] Eichenholtz SN (1966). Charcot joints.

[17] Shibata T, Tada K, Hashizume C (1990). The results of arthrodesis of the ankle for leprotic neuroarthropathy. J Bone Joint Surg Am.

[18] Morrison WB, Ledermann HP (2002). Work-up of the diabetic foot. Radiol Clin North Am.

[19] Morrison WB, Ledermann HP, Schweitzer ME (2001). MR imaging of the diabetic foot. Magn Reson Imaging Clin N Am.

[20] Petrova NL, Foster AV, Edmonds ME (2004). Difference in presentation of Charcot osteoarthropathy in type 1 compared with type 2 diabetes. Diabetes Care.

[21] Ramanujam CL, Facaros Z (2011). An overview of conservative treatment options for diabetic Charcot foot neuroarthropathy. Diabet Foot Ankle.

[22] Pinzur MS, Lio T, Posner M (2006). Treatment of Eichenholtz stage I Charcot foot arthropathy with a weightbearing total contact cast. Foot Ankle Int.

[23] Trepman E, Nihal A, Pinzur MS (2005). Current topics review: Charcot neuroarthropathy of the foot and ankle. Foot Ankle Int.

[24] Brodsky JW, Rouse AM (1993). Exostectomy for symptomatic bony prominences in diabetic Charcot feet. Clin Orthop Relat Res.

[25] Rogers LC, Frykberg RG, Armstrong DG, Boulton AJ, Edmonds M, Van GH (2011). The Charcot foot in diabetes. Diabetes Care.

[26] Jirkovská A, Kasalický P, Boucek P, Hosová J, Skibová J (2001). Calcaneal ultrasonometry in patients with Charcot osteoarthropathy and its relationship with densitometry in the lumbar spine and femoral neck and with markers of bone turnover. Diabet Med.

[27] Young MJ, Marshall A, Adams JE, Selby PL, Boulton AJ (1995). Osteopenia, neurological dysfunction, and the development of Charcot neuroarthropathy. Diabetes Care.

[28] Jeffcoate WJ, Game F, Cavanagh PR (2005). The role of proinflammatory cytokines in the cause of neuropathic osteoarthropathy (acute Charcot foot) in diabetes. Lancet.

[29] Stuck RM, Soh MW, Budiman-Mak E, Lee TA, Weiss KB (2008). Charcot arthropathy risk elevation in the obese diabetic population. Am J Med.

[30] Wukich DK, Sung W (2009). Charcot arthropathy of the foot and ankle: modern concepts and management review. J Diabetes Complications.

[31] Verity S, Sochocki M, Embil JM, Trepman E (2008). Treatment of Charcot foot and ankle with prefabricated walker brace and custom insole. Foot Ankle Surg.

[32] McGill M, Molyneaux L, Bolton T, Ioannou K, Uren R, Yue DK (2000). Response of Charcot's arthropathy to contact casting: assessment by quantitative techniques. Diabetologia.

[33] Sinacore DR (1998). Healing times of diabetic foot ulcers in the presences of fixed deformities of the foot using total contact casting. Foot Ankle Int.

[34] Armstrong DG, Lavery LA, Wu S (2008). Evaluation of removable and irremovable cast walkers in the healing of diabetic foot wounds: a randomized controled trial. Diabetic Care.

[35] Armstrong DG, Short B, Espensen EH (2002). Technique for fabrication for an instant total contact cast for treatment of neuropathic diabetic foot ulcers. J Am Podiatr Med Assoc.

[36] Selby PL, Young MJ, Boult AJ (1994). Bisphosphonates: a new treatment for diabetic Charcot neuroarthropathy?. Diabet Med.

[37] Rogers MJ (2003). New insights into the molecular mechanisms of action of bisphosphonates. Curr Pharm Des.

[38] Jude EB, Selby PL, Burgess J, Lilleystone P, Mawer EB, Page SR (2001). Bisphosphonates in the treatment of Charcot neuroarthropathy: double blinded randomised controlled trial. Diabetologia.

[39] Pitocco D, Ruotolo V, Caputo S, Mancini L, Collina CM, Manto A (2005). Six month treatment with alendronate in acute Charcot neuroarthropathy: a randomized controlled trial. Diabetes Care.

[40] Bern R, Jirkovska A, Fejfarova V, Skibova J, Jude EB (2006). Intranasal calcitonin in the treatment of acute Charcot neuroosteoarthropathy: a randomized controlled trial. Diabetes Care.

[41] Edelson GW, Jensen KL, Kaczynski R (1996). Identifying acute Charcot arthropathy through urinary crosslinked N-telopeptides. Diabetes.

[42] Selby PL, Jude EB, Burgess J (1999). Bone turnover markers in acute Charcot neuroarthropathy. Diabetologia.

[43] Dhawan V, Spratt KF, Pinzur MS, Baumhauer J, Rudicel S, Saltzman CL (2005). Reliability of AOFAS diabetic foot questionnaire in Charcot arthropathy: stability, internal consistency, and measurable difference. Foot Ankle Int.

[44] Mittlmeier T, Klaue K, Haar P, Beck M (2010). Should one consider primary surgical reconstruction in charcot arthropathy of the feet?. Clin Orthop Relat Res.

[45] Farber DC, Juliano PJ, Cvanagh PR, Ulbrecht J, Caputo G (2002). Single stage correction with external fixation of the ulcerated foot in individuals with Charcot neuroarthropathy. Foot Ankle Int.

[46] Pinzur MS (2007). Neutral ring fixation for high-risk nonplantigrade Charcot midfoot deformity. Foot Ankle Int.

[47] Pinzur MS, Sage R, Stuck R, Kaminsky S, Zmuda A (1993). A treatment algorithm for neuropathic (Charcot) midfoot deformity. Foot Ankle Int.

[48] Saltzman CL, Hagy ML, Zimmerman B, Estin M, Cooper R (2005). How effective is intensive nonoperative initial treatment of patients with diabetes and Charcot arthropathy of the feet?. Clin Orthop Relat Res.

[49] Catanzariti AR, Mendicino R, Haverstock B (2000). Ostectomy for diabetic neuroarthropathy involving the midfoot. J Foot Ankle Surg.

[50] Laurinaviciene R, Kirketerp-Moeller K, Holstein PE (2008). Exostectomy for chronic midfoot plantar ulcer in charcot deformity. J Wound Care.

[51] Mueller MJ, Sinacore DR, Hastings MK, Strube MJ, Johnson JE (2003). Effect of Achilles tendon lengthening on neuropathic plantar ulcers. A randomized clinical trial. J Bone Joint Surg Am.

[52] Mueller MJ, Sinacore DR, Hastings MK, Strube MJ, Johnson JE (2004). Impact of Achilles tendon lengthening on functional limitations and perceived disability in people with a neuropathic plantar ulcer. Diabetes Care.

[53] Cooper PS (2002). Aplication of external fixators for management of Charcot deformities of the foot and ankle. Foot Ankle Clin.

[54] Sayner LR, Rosenblum BI, Giurini JM (2003). Elective surgery of the diabetic foot. Clin Podiatr Med Surg.

[55] Papa J, Miyerson M, Girard P (1993). Salvage with arthrodesis, intractable diabetic arthropathy of the foot and ankle. J Bone Joint Surg Am.

[56] Zarutsky E, Rush SM, Schuberth JM (2005). The use of circuler wire external fixation in the treatment of salvage ankle arthrodesis. J Foot Ankle Surg.

[57] Simon SR, Tejwani SG, Wilson DL, Santner TJ, Denniston NL (2000). Arthrodesis as an early alternative to nonoperative management of charcot arthropathy of the diabetic foot. J Bone Joint Surg Am.

[58] Stone NC, Daniels TR (2000). Midfoot and hindfoot arthrodeses in diabetic Charcot arthropathy. Can J Surg.

[59] Pinzur MS (2007). Current concepts review: Charcot arthropathy of the foot and ankle. Foot Ankle Int.

[60] Fabrin J, Larsen K, Holstein PE (2000). Long term follow-up in diabetic Charcot feet with spontaneous onset. Diabetes Care.

[61] Caravaggi CMF, Sganzaroli AB, Galenda P, Balaudo M, Gherardi P, Simonetti D (2012). Long-term follow-up of tibiocalcaneal arthrodesis in diabetic patients with early chronic Charcot osteoarthropathy. J Foot Ankle Surg.

[62] Burns PR, Wukich DK (2008). Surgical reconsturiction of the Charcot rear foot and ankle. Clin Podiatr Med Surg.

[63] Roukis TS, Zgonis T (2004). Postoperative shoe modifications for weight bearing with the Ilizarov external fixation system. J Foot Ankle Surg.

[64] Lowery NJ, Woods JB, Armstrong DG, Wukich DK (2012). Surgical management of Charcot neuroarthropathy – a systematic review. Foot Ankle Int.

[65] Bono JV, Roger DJ, Jacobs RL (1993). Surgical arthrodesis of the neuropathic foot. A salvage procedure. Clin Orthop Relat Res.

[66] Zgonis T (2006). Surgical correction of severe Charcot foot and ankle deformities with external fixation devices. Foot Ankle Q.

[67] Zgonis T, Roukis TS, Frykberg RG (2006). Unstable acute and chronic Charcot's deformity: staged skeletal and soft tissue reconstruction. J Wound Care.

[68] Zgonis T, Roukis TS, Polyzois V (2006). Surgical management of the unstable diabetic Charcot deformity using the Taylor spatial frame. Oper Tech Orthop.

[69] Roukis TS, Zgonis T (2006). The management of acute Charcot fracture-dislocation with a Taylor's spatial external fixation system. Clin Podiatr Med Surg.

[70] Zgonis T, Roukis TS, Lamm B (2007). Charcot foot and ankle reconstruction: current thinking and surgical approaches. Clin Podiatr Med Surg.

[71] Altındaş M, Kiliç A, Ceber M (2012). A new limb-salvaging technique fort he treatment of late stage complicated Charcot foot deformity: two-staged Boyd's operation. Foot and Ankle Surg.

[72] Boyd HB (1939). Amputation of foot with calcaneotibial arthrodesis. J Bone Joint Surg.

